# Sex Dimorphic Responses of the Hypothalamus-Pituitary-Thyroid Axis to Energy Demands and Stress

**DOI:** 10.3389/fendo.2021.746924

**Published:** 2021-10-20

**Authors:** Marco Antonio Parra-Montes de Oca, Israim Sotelo-Rivera, Angélica Gutiérrez-Mata, Jean-Louis Charli, Patricia Joseph-Bravo

**Affiliations:** Departamento de Genética del Desarrollo y Fisiología Molecular, Instituto de Biotecnología, Universidad Nacional Autónoma de México (UNAM), Cuernavaca, Mexico

**Keywords:** sex dimorphism, hypothalamus, pituitary, TRH, energy homeostasis, stress, thyroid, thyrotropin

## Abstract

The hypothalamus-pituitary-thyroid-axis (HPT) is one of the main neuroendocrine axes that control energy expenditure. The activity of hypophysiotropic thyrotropin releasing hormone (TRH) neurons is modulated by nutritional status, energy demands and stress, all of which are sex dependent. Sex dimorphism has been associated with sex steroids whose concentration vary along the life-span, but also to sex chromosomes that define not only sexual characteristics but the expression of relevant genes. In this review we describe sex differences in basal HPT axis activity and in its response to stress and to metabolic challenges in experimental animals at different stages of development, as well as some of the limited information available on humans. Literature review was accomplished by searching in Pubmed under the following words: “sex dimorphic” or “sex differences” or “female” or “women” and “thyrotropin” or “thyroid hormones” or “deiodinases” and “energy homeostasis” or “stress”. The most representative articles were discussed, and to reduce the number of references, selected reviews were cited.

## Introduction

Life evolved through the optimization of multiple pathways that preserve energy homeostasis. The neuroendocrine system of mammals is tuned to sense and react to challenges that disturb homeostasis. Neuronal signals from extrahypothalamic regions, and hormonal signals from the circulation, are transduced by specialized neurons of the hypothalamus, to endocrine signals that control the anterior pituitary output. In the basal state, the hypothalamus-pituitary-thyroid (HPT) axis regulates energy expenditure and in turn, energy status modulates the activity of the HPT axis (1–3). Stress exposure and conditions of negative energy balance as fasting, food restriction (FR) or chronic illness inhibit HPT axis activity, whereas it is activated by energy demanding situations (1, 4–7). The efficient and opportune response of HPT axis to energy demands is crucial to maintain homeostasis.

## 1 Hypothalamus-Pituitary-Thyroid Axis

### 1.1 Elements Involved in the Activity of the HPT Axis

**Figure 1:**
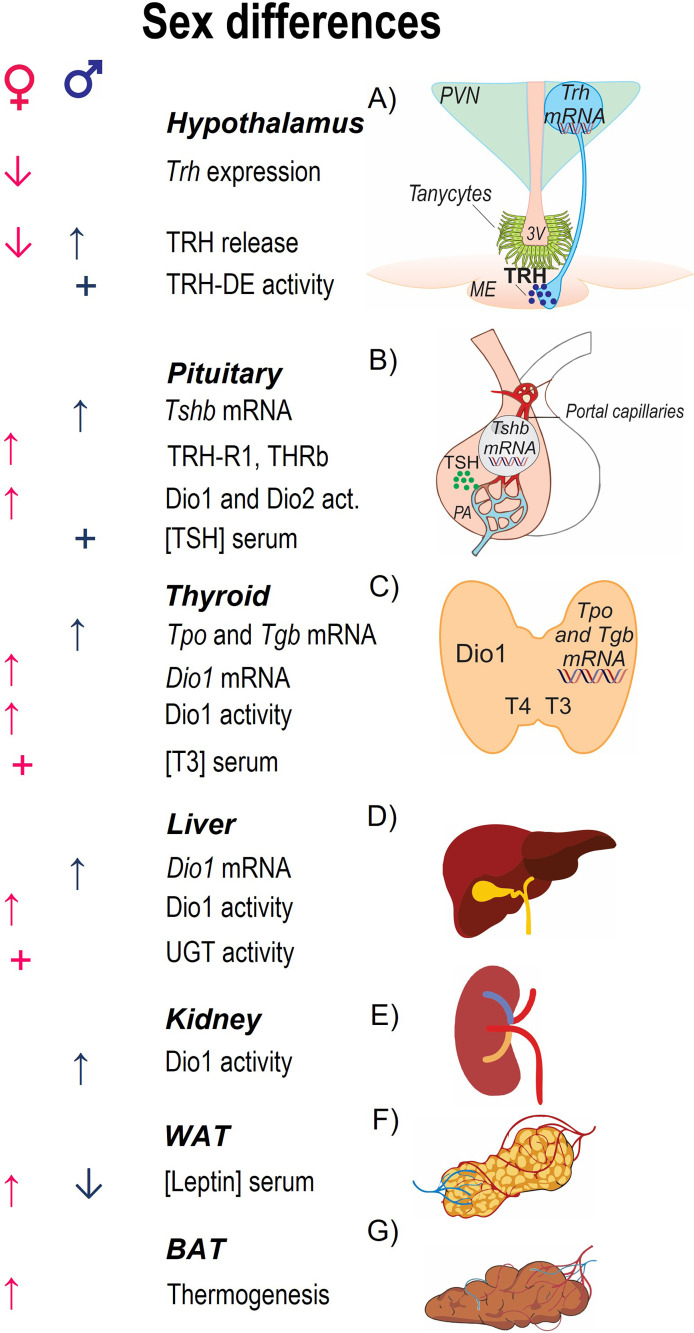
Parameters involved in HPT axis activity and sex differences in basal conditions. The central panel enlists parameters that characterize each region of the axis; **(A)** *Trh* mRNA is expressed in the nuclei of hypophysiotropic cells that reside in the PVN of the hypothalamus, the protein precursor compartmentalized in vesicles that travel to the median eminence (ME) from where TRH is released; at the base of the third ventricle in the ME tanycytes express the TRH-degrading ectoenzyme (TRH-DE). **(B)** At the pituitary, TRH binds to its receptor TRH-R1 in the thyrotropes of the anterior pituitary stimulating the synthesis of the two subunits, TSHα and TSHβ, and TSH release to the portal circulation. **(C)** At the thyroid, TSH stimulates TH synthesis, after uptake of iodine by sodium-iodine symporter it is oxidized by thyroid peroxidase (TPO) and added to tyrosyl residues of thyroglobulin (TGB); TSH also stimulates release of TH (70% T4 and 30% T3). **(D, E)** Circulating T3 is contributed by T4-deiodination by Dio1 in liver and kidney. TH clearance is due to glucuronidation and sulfatation in liver and excretion by kidney. **(F)** Leptin regulates the activity of HPT axis promoting the expression of *Trh* in PVN. **(G)** TH promote facultative thermogenesis inducing the expression of uncoupling proteins in BAT. Arrows indicate stimulation (↑) or inhibition (↓) by sex steroids (testosterone in males; estradiol in females). + indicates that this parameter is greater according to sex.

HPT axis is responsible for the release of thyroid hormones [TH, thyroxine (T4) and 3,5,3’-triiodothyronine (T3)], important participants in energy homeostasis. TH act on multiple cell types and regulate development, growth and function of brain and other tissues through life-span, basal metabolic rate, non-facultative thermogenesis, muscular contraction, energy expenditure, heart rate stimulation, and the expression and activities of many proteins involved in lipid and carbohydrate metabolism (2, 8, 9). The activity of the axis is controlled by the release of thyrotropin-releasing hormone (TRH) from hypophysiotropic neurons localized in the hypothalamic paraventricular nucleus (PVN) that project their axons to the median eminence (ME) and release the processed TRH from their nerve terminals located close to portal vessels and to tanycytes (**Figure 1A**) (1, 3, 5). A TRH-degrading ectoenzyme (TRH-DE or pyroglutamyl peptidase II) present in the membrane of tanycytes may inactivate TRH before it enters the portal vessels; TRH-DE activity is regulated in several *in vivo* situations such as fasting and hyperthyroidism and may be considered a modulator of the quantity of TRH that reaches the anterior pituitary (10, 11). TRH binds to its type 1 receptor (TRH-R1) in the thyrotropes the anterior pituitary stimulating the synthesis and release of thyrotropin (TSH) (**Figure 1B**) (3, 12). Another hypothalamic peptide, somatostatin, inhibits TSH release (13). At the thyroid, TSH binds to its receptor (TSH-R) in follicular cells where it stimulates the synthesis of TH (**Figure 1C**), and the release into the circulation of T4 and a small fraction of T3 (20-30% of TH output) (14). TH are carried in the circulation by blood proteins, thyroxine-binding globulin (TBG), albumin and transthyretin. In humans, TBG binds around 75% of circulating T4 and has higher affinity for T4 binding than for T3; free hormones dictate their activity; only around 0,01% of T4 circulates as free form (15). Rodents lack TBG and use transthyretin or albumin (3). TH enter the cells by the membrane monocarboxylate transporters (MCTs) and organic anionic transport proteins (OATPs). MCT8 and MCT10 transport T4 and T3 in and out of the cells. OATP1C1 is mainly expressed in the blood-brain barrier and transports specifically T4 (16).

TH tissue-concentrations are regulated by the activity of deiodinases (Dio1, 2 and 3) in a tissue-specific manner (17, 18). Dio1 in thyroid, liver, and kidney, regulates the systemic concentration of T3; however, because of its preference for deiodination of T4 inner ring, it produces more of the inactive metabolite 3,3’,5’-triiodothyronine or reverse T3 (rT3) than T3. In hypothalamus, pituitary and target tissues like skeletal muscle, white adipose tissue (WAT) and brown adipose tissue (BAT), Dio2 is responsible of T4 conversion to T3; Dio3 converts T4 to rT3, and T3 to 3,5-diiodothyronine (17, 18). Excretion of TH is catalyzed by UDP-glucuronyltransferases (UGT) located in the liver (19); rodents contain additional conjugating enzymes, sulfotransferases (SULT) (19, 20).

T4 and T3 may bind to membrane proteins of the integrin family (integrin αvβ3) that have greater affinity for T4 (21). T3 is the active hormone in gene regulation; it binds to intracellular receptors that are nuclear transcription factors (THRα and THRβ) which recognize specific consensus sequences of target genes (22). THRβ2 is responsible for T3 negative feedback regulation of *Trh* and *Tsh* expression (12, 22, 23); the mechanisms involved in T3 inhibitory effects on transcription are still in controversy, in contrast to those involved in stimulatory effects (22, 24). THR may form heterodimers with other transcription factors as RXR and modify TRH transcription by different effectors (22, 25, 26). T3 negative feedback effects include other elements of the axis; TH stimulate TRH-DE activity and expression in tanycytes (11), the activity of the two enzymes that can degrade T3, Dio1 and Dio3, whereas they inhibit the expression of Dio2 in pituitary but not in tanycytes (27, 28).

Most work on HPT axis has been performed on male subjects showing its fine regulation that maintains circulating peripheral hormones at a fairly constant range (29). Pathological situations that produce negative energy balance such as starvation or chronic illness inhibit its activity decreasing the expression of *Trh*, and serum concentrations of TSH and T3 (1, 4); chronic stress or corticosterone administration also cause inhibition (5). Energy demands such as cold exposure or increased physical activity stimulate the axis, increasing the expression and release of TRH and TSH within minutes; increased levels of TH may be detected al later times but particularly, TH-induced changes in their target organs (5–7).

Evidence supports in mammals a complex cross talk of intercellular networks and intracellular signaling pathways between TH and sex steroids throughout the life-span of the individual; TH can affect gonadal maturation, steroid synthesis and reproduction (30, 31) as well as the function of the hypothalamus-pituitary-adrenal (HPA) (32). In the following sections we mention the sex differences reported in any of the parameters of HPT axis and on the effects of sex steroids on their regulation. The response of HPT axis to changes in energy homeostasis and stress is discussed in relation to what is known on sex differences; at the beginning of each section a summary of pertinent data on sex dimorphism on energy balance or stress response is be presented.

## 2 Sex Dimorphism of HPT Axis Activity

### 2.1 Basal Conditions

The estrous cycle in rodents lasts approximately four days making it difficult to study female responses (**Supplementary Figure 1**) (33); ovulation occurs during proestrus (12-14 h) with the highest estradiol and progesterone concentrations. Hormones of the HPT axis vary depending on estrous cycle. *Trh* expression in hypothalamus is high in the later diestrus phase, tends to decrease during proestrus and early estrus (34). Serum T4 and T3 concentrations increase during proestrus (35). Thiouracil-induced hypothyroid-female rats have irregular estrous cycles after 16 days of treatment, they show a prolonged diestrus phase, lower serum estradiol but higher progesterone concentrations during proestrus compared to the same phase in euthyroid rats and a peak of serum T3 and of T4 concentrations at proestrus (36).

The concentrations of serum TSH and TH concentrations differ between male and female rodents (37, 38), as well as the expression of various elements responsible of the basal activity of HPT axis (**Figure 1** and **Supplementary Table 1**). No sex difference has been reported on *Trh* mRNA levels of PVN or TRH content in the median eminence of adult rats (39, 40). Pituitary *Tshb* mRNA levels, as well as TSH concentration in this gland and serum TSH are higher in males than females (37, 39), probably due to testosterone induced *Tshb* mRNA expression in pituitary of intact and gonadectomized male rodents (41, 42), to the inhibitory effect of estradiol and to the higher concentration of T3 and THR in pituitary of females (43). Although TSH concentration is higher in males than females, TRH-induced release of TSH is not sex-dependent (37), likely due to the augmented sensitivity of female pituitary cells to TRH by estradiol-induced increased expression of pituitary TRH receptor (44). T4 circulating concentration does not differ between male and female rodents, but that of T3 increases after puberty and is higher in females than males (38, 45). T3 circulating concentration is mainly regulated by Dio1 produced in liver and kidney and although Dio1 activity in these organs is greater in males than females, it produces mainly rT3 (38). Transthyretin transports TH in blood of rodents, its expression is increased in liver by estrogens and androgens (46). TH clearance is also sex-regulated; the activity of bilirubin-UGT is greater in females than males in basal conditions, without difference in the activity of androsterone-UGT (47).

In humans, research on HPT axis physiology has also been performed preferentially in men, although women present higher incidence of its disfunction than men (48, 49). Sex dimorphism in serum TSH concentration in humans varies with age and health state. Serum FT4 and FT3 concentrations in men are greater than in women (50–53). Estrogen increases the concentration of TBG in the circulation, whereas glucocorticoids diminish it, making TBG concentration another point of regulation; for example, in excess concentrations of estrogens as with usage of contraceptive drugs (54). Before menopause, there are no differences between sexes in serum TSH concentration of healthy adult humans (50, 55), whereas after menopause it is higher in women than in men (56). Furthermore, women with metabolic syndrome have higher TSH concentration than men with this condition (51–53).

### 2.2 Effect of Castration and Hormone Replacement

*In vivo* effects of sex steroids have been evaluated by direct administration or after gonadectomy (**Supplementary Table 1**). Ovariectomy (OVX) increases body weight and food intake (57, 58). *Trh* expression in hypophysiotropic neurons is increased in OVX rats compared to values at diestrus that are attained with 50 μg/kg of estrogen replacement and decreased with 100 μg/kg (57). Compared with intact rats, hypothalamic *Dio2* expression is decreased in OVX rats (58) which, if it corresponds to the activity in tanycytes, would decrease the T3 feedback effects consistent with increased expression of hypothalamic *Trh* after OVX. Estradiol treatment at 25 μg/kg does not increase TRH concentration in hypophysial portal system of OVX rats, but it does if combined with progesterone treatment (10 or 50 mg/kg) (59). OVX increases the mRNA levels of the α and β subunits of *Tsh* in pituitary (60) but not TSH serum concentration unless replaced with 50 μg/kg estrogen (38, 57). Dio1 activity in pituitary is not affected by OVX though increased by estradiol treatment (7 μg/kg) and attenuated by progesterone (60). Serum T4 concentration does not change by OVX but is slightly inhibited with low concentrations of estrogen replacement (1.4 μg/kg) (38). The effect of OVX on serum concentration of T3 is controversial, from no effect (57) to a slight or strong decrease (38, 61), increased only with replacement with 100 μg/kg and higher doses of estrogen (57); however, combined estradiol and progesterone treatment increases T3 serum concentration and decreases T4 concentration in OVX rats (58, 62). At the thyroid level, OVX decreases the density of TSH receptors that is normalized after estradiol replacement (61, 63); estradiol injected in normal (64) or OVX rats increases iodine uptake and the activity of TPO, which would favor an increase in T3 synthesis (61); progesterone injection inhibits the stimulatory effect of estradiol on iodine uptake in normal rats (64). TPO activity uses H_2_O_2_ as substrate; the stimulatory effect of estrogen on TPO activity and on the expression of enzymes involved in the formation of H_2_O_2_ may increase the oxidative state of the thyroid and explain the higher susceptibly to thyroid disfunction in females than in males, including higher incidence of thyroid cancer (65).

In contrast, orchidectomy decreases serum TSH concentration which is normalized by testosterone but not by estrogen treatment of male rats (66). Castration in male rats increases Dio1 activity in pituitary, and testosterone treatment (4 mg/kg) does not reverse this effect (60). No effects on T4 or T3 serum concentrations are detected in orchiectomized males, treated or not with testosterone or estradiol (66).

Sex steroids influence thyroid function also in humans; hyperestrogenemia caused by contraceptives, hormone replacement or ovarian hyperstimulation increases serum TBG and decreases FT4 levels inducing hypothyroidism (67). In transsexual male-to-female patients, treatment with oral estrogens increases serum concentrations of TBG, TT4 and TT3, while in female-to-male patients treated with testosterone, serum TBG and TT4 concentrations decrease (68).

### 2.3 Sex Dimorphism in HPT Axis Disfunction

Few animal studies have compared both sexes within the same experimental design. In hypo- or hyper-thyroid treatment of male and female mice of the same age (hypothyroidism: low iodine diet and 0.02% methimazole/0.5% sodium perchlorate in drinking water; hyperthyroidism: 1 mg/kg T4 *via* intraperitoneal) produces some of the expected results though differently in males or females; hypothyroid male mice have high serum cholesterol concentration whereas hyperthyroid females have increased triglyceride serum concentration, increased food intake and body weight gain; after 7 weeks of the respective treatments, serum concentrations of TT4, FT4, and FT3 are higher in hyperthyroid females than in males and these differences augment with age (5, 12, 24 months old) (69, 70). Hypothyroid males lose body weight and are hypothermic though their decrease in serum TT4 concentration is like that of females; furthermore, serum FT4 concentration decreases only in old female rats and that of FT3 remains normal. Body temperature of females is higher than in males, irrespective of thyroid status, due to estrogen effects (71). Whether the sex differences detected in the serum TH concentrations are due to differences in deiodinases or clearance events remains to be clarified (20, 47). For example, of the three UGT isoenzymes, hepatic bilirubin-UGT activity which is higher in females increases by FR more in males than in females (47); activities of SULT are regulated in a sex dimorphic manner by age (20). The expression of various TH-target genes in BAT, heart and liver is differentially modified by thyroid status and sex. Behavioral tests confirm a higher locomotor activity in females, that increases in hyperthyroidism, as well as a better motor coordination but diminished muscle strength, while hyperthyroid males have better coordination in the motor rod (69, 70); these effects were attributed to female muscles being more resistant to fatigue than those of males (72). The latter changes reported in males coincide with previous reports in the literature (73); thus, females seem less affected despite having a dysregulation on their TH levels. Unfortunately, mice were housed in different conditions (males in individual cages and females in group/cage); because isolation is a stressful factor that induces hypothyroidism in male mice and differentially alters various parameters depending on sex (74, 75), the validity of some of these sex differences needs to be established.

The prevalence of thyroid disorders is higher in women than men (76, 77). Hypothyroidism is diagnosed by serum concentration of TSH above normal (>4.5 mU/L) and that of FT4 below (primary hypothyroidism), although tertiary and secondary hypothyroidism may occur when deficits are at hypothalamic or pituitary levels and both serum TSH and TH concentrations are low (78, 79). Hypothyroidism reduces metabolic rate, and some symptoms include fatigue, body weight gain, hypothermia, dry hair and skin, cardiovascular risk factors, dyslipidemia and increased atherosclerosis. It is estimated that hypothyroidism frequency may be up to 15% with a large percent of undiagnosed subclinical hypothyroidism that increases with age (78). In an extensive US survey, no differences were found between male and female serum TSH concentration, except with women over 50 years old whose values were higher than those of males (80); central hypothyroidism occurs rarely (<0.1%) (79). A close association of hypothyroidism with symptoms of metabolic syndrome has been now recognized (52, 81, 82), women with metabolic syndrome having higher serum TSH concentration than men with this condition (51–53). Hyperthyroidism (overt and subclinical) produces low BMI, waist circumference and blood triglyceride concentration, but high blood pressure and serum high-density lipoprotein cholesterol concentration compared with euthyroid men. Women do not present symptoms of hyperthyroidism (53, 81, 82). Men with subclinical hypothyroidism have higher serum triglyceride concentration and abdominal fat weight than women, while subclinical hypothyroid women are more propense to become obese (81, 82), an effect increased after menopause (51–53). An extensive study performed in China demonstrates that obese women have a higher risk to suffer overt and subclinical hypothyroidism (22.7% and 22.1% respectively) than non-obese women, while in men there is no association between obesity and hypothyroidism (83).

TH are required, not only for the adequate development of central nervous system but also for its adequate function throughout life, which explains why hypothyroidism can affect mood and behavior, cognition, memory, visual attention and motor skills (9, 84). Thyroid deficiency during development affects the adequate development of brain which, depending on the stage of development, may induce several diseases as for example affect neural circuitry and cause autism observed more in boys. Neuropsychiatric disorders that are related to thyroid disfunction (more frequently affected in women) coincide with their higher incidence, for example, TH or TSH serum levels associate with Alzheimer disease in women (85). Subclinical hypothyroidism has been related with psychiatric disorders such as anxiety and depression (85). Few studies have associated sex differences in depressive patients with subclinical hypothyroidism; however, a recent study reports its prevalence in women with depression being approximately two times higher than in men (86).

In humans, the main cause of primary hypothyroidism in many countries is iodine deficiency, whereas in iodine-sufficient countries is Hashimoto’s disease, although hypothyroidism is increasing now due to pollutants known as endocrine disruptors (83, 87). Grave’s disease (GD) and Hashimoto’s thyroiditis (HT) are of autoimmune origin, preponderant in women (83). GD corresponds to 70-80% of hyperthyroid patients, in a ratio of 8:1 of women to men; GD is due to auto-reactive antibodies against TSH-R that signal as TSH in thyroid causing hypersecretion of thyroid hormones (83, 87). HT is caused by the presence of antibodies against TPO and TGB, as well as infiltration of lymphocytes in thyroid (88); HT has also a higher incidence in women 10-25:1 (87, 88). These pathologies are part of the autoimmune thyroid disorders that show changes in methylation of several genes, gene polymorphisms and also miRNAs identified either specific for each disease or common to both (89). However, the high preponderance in women of this diseases and in many other autoimmune ones, as well as in experimental animal models, support that the sexual dimorphism in normal and abnormal immune responses could be due to epigenetic alterations in X chromosome (**Box 1**) which codes for many genes of in the immune system (83, 98, 99). Common features among several autoimmune diseases involve incomplete inactivation of some genes in the X chromosome (XCI), or asymmetric inactivation of X chromosome (skewed XCI) where 75-80 or over 95% of one parent’s chromosome is inactivated (98, 99). Analyses of 309 GD and 490 HT female subjects together with meta-analyses of previous reports confirm the increased XCI skewing and extreme skewing in female subjects with GD and HT respectively (100).

**Box 1 The role of sex chromosomes in metabolic sex dimorphism.**
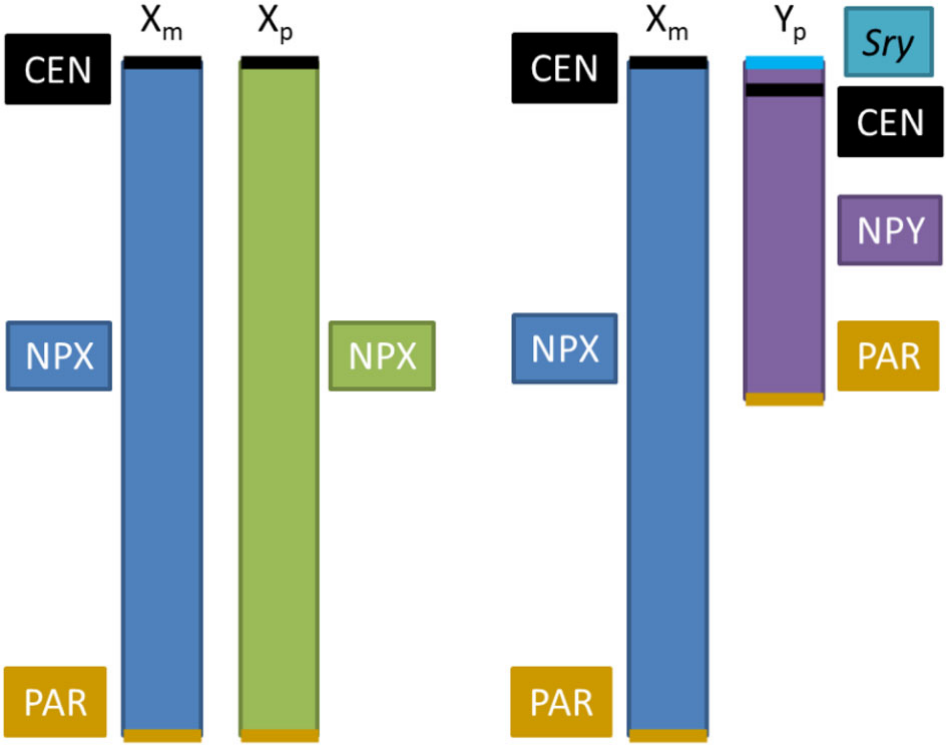
Sex dimorphism in behavior and physiology in mammals originates in the hormonal differences between both sexes and albeit not thoroughly identified, to genes located on the X and Y chromosomes (90, 91). X and Y chromosome genes may be expressed at different levels in XX and XY non-gonadal cells, producing effects independent of sex hormones (92). X and Y chromosomes contain a set of genes disposed in pseudo-autosomal regions (PAR) that are implicated in critical cellular functions, such as control of chromatin modifications, transcription, translation, RNA splicing, protein ubiquitination, etc. (91). Aside from the PARs, the genomic dosage is different in males and females, since females have two copies of non-PAR region of the X chromosome (NPX), whereas males have one NPX and one non-PAR region of Y (NPY) (92). This sex unbalance of X chromosome number is equalized by 2 mechanisms: 1) Upregulation of X genes in males, and 2) silencing of one X chromosome in females. Upregulation of X genes involves increases in transcription (enhanced H4K16 acetylation, or enrichment of RNA polymerase II at the 5’-end of X genes), RNA stability (longer half-life), and translation (a greater number of ribosomes) [reviewed in (93)]. X chromosome silencing is produced by *Xist*, a long non-coding RNA that coats X chromosome and recruits protein complexes to implement gene repression; this also includes epigenetic changes, such as recruitment of polycomb repressive complexes to implement repressive histone modification, followed by DNA methylation at CpG islands to stabilize silencing (94). Since females have a X chromosome from the mother (X_m_) and a X chromosome from the father (X_p_), X chromosome silencing is random in somatic tissues, producing a mosaicism that leads to less phenotypic variability among females than males and to average differences in phenotype between sexes (91, 92). Evidence that sex chromosomes influence programming of metabolic regulation and how they interact with sex hormones have been obtained from a model that generates mice with four combinations of gonads-sex chromosome: XX mice with female gonads, XY mice with male gonads, XX mice with male gonads and XY mice with female gonads; this model allows to evaluate the influence of sex chromosomes (90, 95). The dosage of X chromosomes contributes to an accumulation of fat and an increase of food intake; however, the distribution of fat is different between genotypes independent of gonadal sex, XX mice having more subcutaneous fat whereas XY mice more visceral fat (96).

## 3 The HPT Axis in the Symphony of Energy Homeostasis

Energy balance depends on food intake and/or metabolism of endogenous reserves (glycogen and fat depots), and on energy expenditure; these events differ between males and females (95, 96), are regulated by sex steroids, and also on sex chromosomes (**Box 1**) such as the higher expression of leptin in subcutaneous fat of females before puberty or the sex-differences in fat distribution. Higher proportion of subcutaneous fat in females makes them more resistant to pathologies associated with the metabolic syndrome than males, as it produces leptin and adiponectin; estradiol stimulates while testosterone inhibits leptin expression (101). Estrogen regulates energy expenditure, body weight, fat distribution, and appetite in mice; it also suppresses WAT accumulation by decreasing fatty acid and triglyceride synthesis or lipogenesis (96). Testosterone exerts similar effects when aromatized to estrogen but also increases lipolysis in visceral WAT through the interaction with its receptor, but the exact mechanism is not yet determined (96).

**Figure 2:**
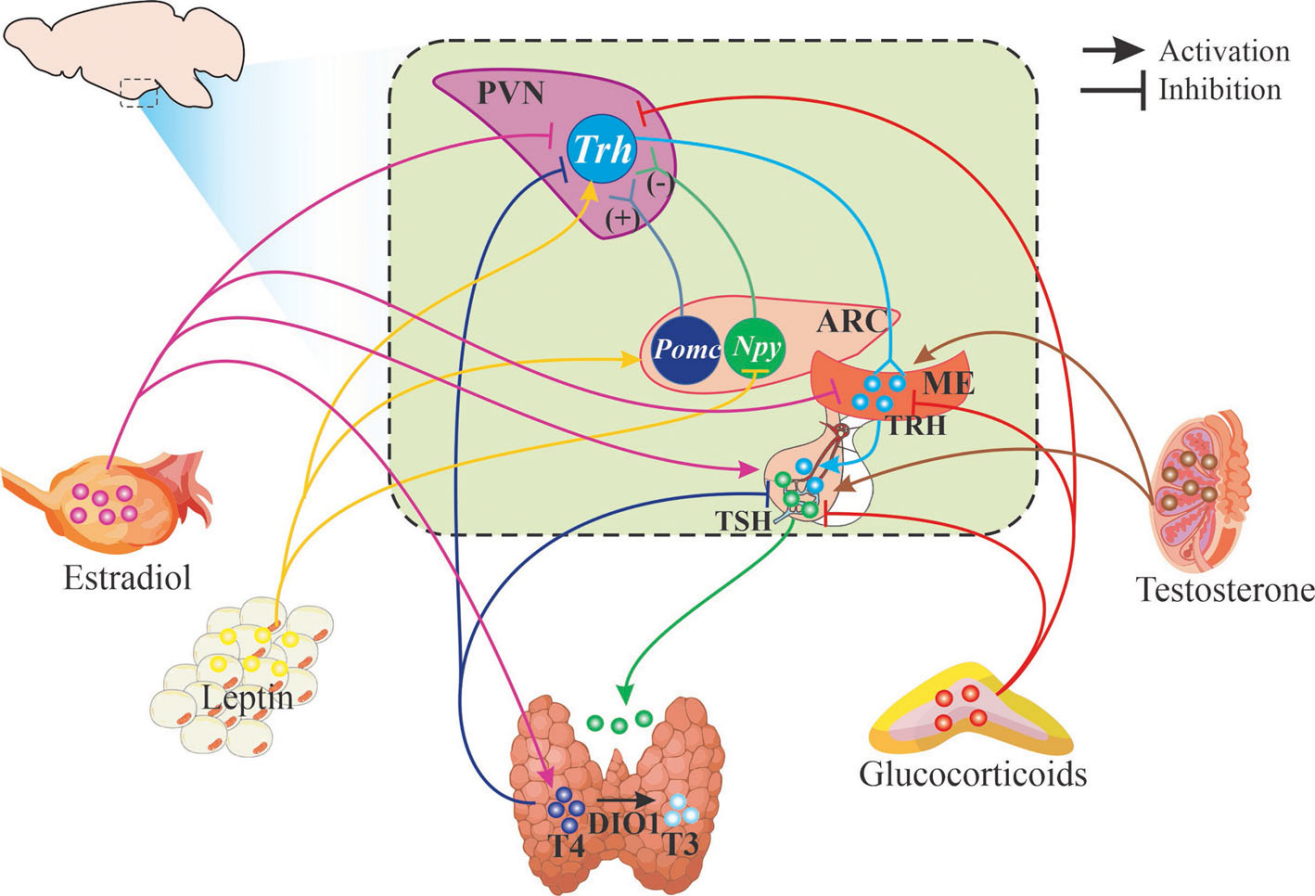
Schematic representation of HPT axis regulation. Figure illustrates hypophysiotropic TRH neurons of the paraventricular nucleus of the hypothalamus (PVN) that send their axons (light blue line) to the median eminence (ME) where they release TRH. Only known effectors are signaled: neurons from the arcuate nucleus that synthesize POMC or NPY stimulate or inhibit *Trh* expression and release. Arrows (light blue and light green) depict the various steps of the HPT axis that are regulated by different hormones at the level of synthesis or release of TRH, TSH and thyroid hormones as well as their receptors (details in text).

TH regulate energy expenditure and many metabolic processes such as lipid metabolism promoting lipolysis as well as lipogenesis in adipose tissues (2, 8); in turn, the activity of the axis is modulated by nutrient status and energy demands (**Figure 2**). Nutrient status is signaled to TRH hypophysiotropic neurons by neurons from the arcuate nucleus (Arc) that integrate peripheral signals from circulating hormones, such as leptin and insulin, and express the anorexigenic pro-opiomelanocortin (POMC), precursor of α-Melanocyte-stimulating hormone (α-MSH) which is released in PVN and interacts with the type 4 melanocortin receptor (MC4R) that induces *Trh* gene transcription (102–104). MC4R receptor is downregulated by TH contributing to T3 feedback regulation of HPT axis (105). Conversely, orexigenic peptides AgRP and NPY inhibit the expression of *Trh* in PVN (1). Although estrogen stimulates *Pomc* expression and is postulated to mediate some of the anorexic effects (106), the role of PVN-MC4R was recently discarded (107); this explains why estrogen effect on *Trh* expression is not stimulatory as would be expected if the POMC-neurons that innervate TRH neurons were activated and supports the heterogeneity of POMC neurons in the Arc (108). Besides the indirect stimulatory role of leptin on the synthesis of TRH through α-MSH released by POMC neurons and stimulating pCREB (in 66% of *Trh*-expressing cells in mid PVN), it has direct stimulatory effects on TRHergic neurons activating its receptor Lep-Rb inducing the phosphorylation of Janus kinase 2 that in turn phosphorylates signal transducer and activator of transcription 3 (in 28% of cells) allowing its binding to *Trh* promoter (104, 109, 110).

### 3.1 Sex Differences in the Response of HPT to Positive or Negative Energy Balance

The combined action of TH and sex steroids on metabolism may explain some of the sex differences of HPT activity. Conditions of energy deficit due either to FR or to food deprivation inhibit the HPT axis at multiple levels (1, 111); PVN-*Trh* expression and serum concentrations of TSH and TH decrease more in puber female than male rats (39), whereas inhibition occurs faster and stronger in adult males than in females (40). PVN-*Trh* mRNA levels and TSH, T4 and T3 serum concentrations decrease after 24 h of fasting in male rats while in females it takes 48 h to affect *Trh* expression and serum T3 concentration; serum corticosterone concentration is higher in females than males since the first day of fasting (40). Females reduce more retroperitoneal WAT mass than males after 24 or 48 h of fasting, consistent with other reports and with the preference for fat usage in females compared to males, which permits them to preserve protein and lean-mass (40, 101). Females are also more resistant to diminished HPT axis activity after two weeks of food restriction; even less than 20% decrease in food intake in males diminishes *Trh* expression in PVN and serum concentrations of TSH and leptin, whereas no changes are observed in females with reduced food intake of 30%, though females reduce leptin serum concentration almost as males do (112). These results suggest that either food deprivation or restriction leads to decrease serum T3 concentration and body or fat weight loss, and that in all conditions of negative energy balance the diminution of HPT axis activity is higher in males than in females.

In cases of energy excess provoked by high saturated-fat diet (HFD), the HPT axis is activated in male rats and mice (111, 113), but few reports have evaluated male and female rodents simultaneously. HFD (with saturated and monounsaturated fatty acids) for 40 days starting at weaning increases 2x leptin serum concentration and decreases the ratio of TT4/TT3 in male rats whereas in females, leptin concentration increases 3x with no changes in the ratio of TT4/TT3 (114). In contrast, if a palatable diet high in unsaturated fat and carbohydrates (HFCD) is available for free choice with chow, during six weeks starting at adulthood, females consume more palatable diet and increase more their relative fat weight than males. Serum concentration of insulin increase in both sexes but only in females there is an increase of leptin; levels of *Pomc* mRNA in mediobasal hypothalamus (MBH) and serum T4 concentration augment in females whereas serum TSH concentration diminishes in males likely due to increased ME *Trhde* expression (115). Several reports using male rats or mice and a high fat diet rich in unsaturated fats show increased HPT axis activity related to the obese state (115, 116). The differences observed depending on the type of fat used and the proportion of carbohydrates require further analyses.

In euthyroid humans, studies on the relationship between circulating concentrations of TSH and free TH with obesity have provided controversial results; in a recent study (117) with over 586 women and 437 men where several metabolic parameters were measured and classified according to sex, age, BMI, % of body fat and metabolic phenotype (metabolically healthy non-obese, metabolically healthy obese and metabolically unhealthy obese) showed that serum FT3 concentration and FT3/T4 increase in obese, whether healthy or unhealthy, and these values are positively related in women to % of fat but not to BMI; serum T4 concentration correlated negatively with the unhealthy obese phenotype, and serum TSH positively, suggesting they contribute to the metabolic dysregulation and not the % of fat (117).

### 3.2 HPT Axis Response to Energy Demands in Adults

Cold exposure rapidly activates the HPT axis in males, consistent with the role of TH in thermogenesis increasing synthesis of uncoupling proteins in BAT and in muscle, as well as the expression of adrenergic receptors (6, 118). Male rats can maintain their body temperature after 1-2h of cold exposure; females in contrast do not if they are in diestrus phase but do it if in proestrus; in OVX rats body temperature is reduced more rapidly than in control rats, an effect which is prevented if rats are injected with estradiol (71, 119). Cold exposure rapidly stimulates *Trh* expression in PVN and serum TSH concentration after 1 h in male rats while not in virgin or lactating females (5, 6, 120) unless they are ovariectomized (57). Estrogen administration diminishes temperature loss by increasing vasoconstriction and heat production, by stimulating the medial preoptic area and dorsomedial nucleus involved in the sympathetic activation of BAT and muscle (71, 120, 121). BAT activity is enhanced by estrogens at various levels: expression and sensitivity of β3-adrenergic receptors, density and differentiation of mitochondria, and together with progesterone the expression of *Ucp1* (96, 101, 120). The thermogenic effect of estrogens seems to cancel the cold stimulation of HPT axis activity in females explaining the high diversity observed in randomly cycling rats.

Physical activity and exercise require energy provided by the adequate mobilization of fuels to active tissues as heart and muscle (8, 122, 123). Data on HPT axis response after exercise has been controversial since conditions such as exhaustion, that induce a negative energy balance or high stress, inhibit the axis (124). Voluntary exercise performed in a running wheel does not cause stress and instead, diminishes HPA axis responses to stressors (125). Females run at least twice more than males, but loose a similar amount of fat mass; exercise activates the HPT axis in both sexes at different levels; *Trh* expression increases in PVN of male rats without affecting serum TH concentration measured 3 h after the last exercise boot, while in females *Trh* expression does not change but TSH concentration increases. In both sexes, *Trh* mRNA levels or serum concentrations of TSH, T4 or T3 correlate positively with the amount of distance ran, and negatively with WAT weight (7, 75, 112). These results indicate that females require much more exercise than males to lose the same amount of fat and to activate the HPT axis; females do not lose body weight as males do, explained by males’ loss of lean mass (126).

The effect of exercise on HPT axis activity in humans varies with its duration; the increase of serum TSH, T4 and T3 concentrations during exercise are reverted during recovery (127). Depending on health state and physical condition, the response of HPT axis to exercise differs between men and women. Untrained young adult men show no changes in serum TT4 and TT3 concentrations after an aerobic exercise period (running 4-6 miles each 2 days per 6 weeks), but serum TT4, TT3 and FT3 concentrations diminish after extenuating exercise (128, 129). In the case of athletes, T3 and T4 serum concentrations decrease in trained men compared with untrained, while athlete women reduce serum T3 concentration only when they present amenorrhea (130). Exercise conditions that induce negative energy balance decrease serum TH concentrations, consistent with the tight regulation of HPT axis with even small amounts of food restriction. When fat mass is considerably reduced, serum leptin concentration diminishes, which most probably lowers PVN TRH neurons activity and TSH release (1, 58, 104, 109, 110).

## 4 Stress and HPT Axis

As mentioned in the preceding section, estrogen and TH participate in energy homeostasis regulating food intake and metabolism of lipid, carbohydrate and protein as well as the flux of fuels to active tissues (8, 123). Stress is another important player; acute stress activates the sympathetic nervous system and the HPA axis that mobilize energy substrates by stimulating lipolysis in WAT and increase the circulating concentration of glucose (131). Chronic stress in contrast promotes the accumulation of fat specially in the visceral region by the local activation of glucocorticoids (GC) induced by 11β-hydroxysteroid dehydrogenase-1, favoring hypertrophy of the adipocytes and altering responses of the HPA axis to subsequent stressors (131, 132). Responses to stress are sex-dimorphic; adult female rodents have higher serum corticosterone concentration in basal condition or after acute stress exposure than males; this difference is attributed to a stimulatory effect of estrogens at all levels of HPA axis, and to a slower feedback inhibition due to a lower expression of glucocorticoid receptor (GR) in PVN and pituitary in females than males; androgens in turn, inhibit HPA axis activity at different levels (133). Stress response in humans is also sex dimorphic; women have higher circulating concentration of cortisol and stronger HPA axis stress-responses than men since estrogens stimulate, and androgens inhibit HPA response; women are more susceptible to adrenocortical disorders (134).

### 4.1 Effects of Stress on HPT Axis Activity in Adults

Although in males several types of acute or chronic stress inhibit HPT activity (5), there is limited work on female subjects. Psychological stressors as immobilization reduces *Trh* expression in PVN concentration in young adult rats independent of sex, plasma TSH to a higher extent in males, that of FT3 decreases only in females who show a higher increase in serum corticosterone concentrations; this sex difference is not perceptible in old rats (135, 136). Chronic stress imposed by individual housing (isolation) for 7 days after weaning increases serum TT4 concentration in female but not in male rats (114). Chronic variable stress, a model that resembles chronic stress in humans as it produces a constant secretion of glucocorticoids, increases *Tshb* expression in pituitary of females but not male mice (137) while in rats, TSH serum concentration decreases in males (138). This contradiction could stem from the type of stressors applied or to species-differences (139) and illustrate the difficulty in pointing the causes of sex-differences. The effect of physical stressors such as pain or electric shocks, which diminish HPT activity in males, has not been studied in females (5). Cold exposure and exercise have also been considered physical stressors as they activate the HPA however, they also activate HPT axis (5).

Not only basal activity of HPT axis is altered by stress, the stress history of the animal also affects HPT axis response to energy demands. Albeit not studied yet in females, acute or chronic stress blunts the acute cold-induced stimulation of PVN *Trh* synthesis, TSH release and BAT activation in male rats (6, 138), through a direct effect of corticosterone inhibiting cold-induced CREB phosphorylation in TRHergic neurons (140). Some types of stress can interfere with the stimulatory effect of voluntary exercise on HPT axis activity and loss of fat mass; male or female rats are similarly affected by two weeks of chronic intermittent restraint but in response to isolation stressed males do not lose fat nor activate any of the variables of the HPT axis whereas females are not affected (75). These latter results add up to the results of females’ response to fasting (40) that strongly suggest HPT of adult females to be less susceptible to change.

### 4.2 HPT Axis and Stress Through Development

No sex differences in the expression of HPT components have been reported before puberty, with the exception that female rats have a higher TSH serum concentration in the first days of birth compared with males (141). Sex differences in physiology may be triggered by external influences along the life-span of the animal. Glucocorticoid excess during development causes sex-specific metabolic mis-programming (142); it is thus feasible that HPT axis development may also be affected. HPT axis ontogeny occurs before and after birth (**Supplementary Figure 2**), including hormonal synthesis and secretion, and functionality of hormone receptors, thus its vulnerability to epigenetic changes may relate to the timing of insult exposure (143).

#### 4.2.1 Prenatal Life Stress

During gestation, embryos depend on the hormonal milieu of the mother for an adequate development of organs and tissues, especially the brain; her nutrition, or exposure to drugs and toxins, can affect the developmental programming of the offspring (143). TH are essential in brain development, so any event that diminishes their production or signaling in the mother such as exposure to GC, stressors, toxins, or nutrient unbalance during development causes many deleterious effects in offspring brain such as decreased cognition (9, 111, 144); again, few reports studied differences according to sex. TSH plasma concentration is higher in female rat offspring of mothers that consume alcohol or glucose during lactation than male (145). At adulthood, male offspring of mothers that consume a high fat diet for 8 weeks (before mating, during gestation and lactation) express less *Trh* in PVN than females, whereas *Dio2* expression in hypothalamus is lower in female offspring (146). A high exposure to GC before birth alters the activity of HPT axis at adulthood; in female rats, body temperature decreases at estrus and diestrus stages, which may be related to decreased *Trh* expression in PVN and T3 serum concentration; male rats only have low T3 serum concentration (147).

Environmental contaminants derived from industrial products of diverse use as insecticides, fertilizers or plastics (such as phthalates and bisphenols) interfere with thyroid hormone signaling because they are recognized by thyroid hormone receptors or affect TH synthesis and metabolism (79, 144, 148); they affect also steroid synthesis or steroid-hormone receptors (149). Bisphenols and phthalates act at low concentrations, categorized as endocrine disruptors, and are particularly dangerous in a pregnant woman as they interfere with the effects of TH on growth and development. Sex dimorphic effects have been identified for these compounds, particularly in neurobehavior, albeit it might be difficult to distinguish cause from effect on sex dimorphic responses in thyroid or steroid hormones when humans are exposed to mixtures of contaminants (144, 148, 149). Few studies report sex differences on the effects of endocrine disruptors on elements of the HPT axis; exposure to phthalates during pregnancy decreases FT4 serum concentration mainly in girls (150).

#### 4.4.2 Neonatal Life Stress

Several models of early life stress have been developed to study the deleterious effects of stress during the neonatal period (151). A well characterized model that does not cause physical harm or many hours of fasting is maternal separation (MS) during lactation; pups are separated 3 h or less from their mother (152) and the reduced leaking and grooming diminish serotoninergic input to the hippocampus decrease GR expression up to adulthood that decreases hippocampal feedback inhibition on PVN *Crh* expression causing HPA axis hyperactive response to stress (153). MS disturbs the neural programming of the offspring producing long term effects on its behavior and stress responses, many of which show sex dimorphism (154).

MS alters basal parameters that set HPT basal activity; at adulthood, MS-females gain body and WAT weight and increase *Trh* expression in PVN more than non-handled (NH) females. MS males express more *Trhde* in MBH, have higher corticosterone and lower TSH and T3 serum concentrations than NH controls and present a blunted response to 48h of fasting (155). *Trhde* expression is increased after HFCD intake only MS male rats who were not protected from an acute stress as controls or females on diet or chow (115); HFCD does not protect from stress-induced inhibition in PVN *Trh* expression in males of NH or MS groups while T3 serum concentration decreases in MS in contrast to the lack of response of females (115). Thus, maternal separation affects the basal activity of HPT axis in both males and females but only in males affects HPT response to metabolic challenges as fasting and hypercaloric diets (115, 155).

The response of the HPT axis to exercise in MS animals differs from that observed in chronically stressed animals. While chronic stress in adults inhibits HPT axis activity and fat-loss responses to voluntary exercise in males, more than in females (75), the opposite is observed after maternal separation. As adults, MS males respond as controls do: same loss of fat mass (albeit leptin serum concentration is lower in MS than in NH), PVN *Trh* levels and serum TH concentrations correlate positively with distance, and negatively with fat mass. In contrast, running is reduced in MS females, do not lose fat mass and have no significant correlations to those seen in NH (112). MS-induced susceptibility to stress and metabolic disfunction is reduced by the beneficial effects of exercise on attenuating stress responses in males but not in females which contrasts the enhanced susceptibility of MS males after fasting (112, 155). Epigenetic changes in the PVN were detected on the proximal rat *Trh* gene-promoter; we hypothesized that CRE site hyper methylatation would inhibit the effect of neuronal signals from NA or POMC neurons that increase *Trh* expression due to the binding of pCREB to the promoter (156). No differences in percent of methylation at CRE site are detected, instead a CpG next to site-4 [−59/−52 (157)] is hypermethylated in females, and CpG in site-5 hypomethylated in males; these are regions of THR binding, site-4 has been postulated as a response element for thyroid hormones (158). Hypermethylation will diminish T3 feedback effect on *Trh* transcription, coincident with the higher expression of *Trh* in PVN of MS-female rats and their slower response in conditions as fasting (155).

Evidence accumulates now, especially in males, that stress affects metabolism and cause HPT axis disfunction (5). In some cases, effects may perdure for a long time; however, stressor effects cannot be generalized by sex or age, and this is just beginning to be understood. Knowledge of the neuronal circuits involved according to the type of stressor may eventually lead to its control. The ability to cope with stress, called resilience, is observed in males submitted to neonatal stress and then exercise but still seems to vary depending on the type of exercise and type of stress. Rats have shown stress resilience whether exercise is performed before or after stressor exposure; in certain types of stress, females respond faster than males (159). Our results that stress curtails adequate lipolysis as it impedes exercise-induced fat loss and diminishes running probably due to fatigue, suggest interference with the fast and intermittent activation necessary for TH to exert their metabolic effects needed to supply fuels to oxidative tissues (8, 122, 123). These effects, together with the blunting effect of stress on cold-induced activation of HPT axis that impedes to control body temperature (138) shown in adult males, may contribute to the symptoms of subclinical hypothyroidism. The susceptibility to stress effects depends on sex, age and type of stress.

## 5 Conclusions

We reviewed the different physiological aspects that play a role in defining sex dimorphism in HPT axis activity, due not only to sex steroids but also by the change of diet, sedentarism, and stress, whose impact differs among sexes. Sex dimorphism in HPT axis activity may be due to sex steroids influence in a direct or indirect manner, causing different responses depending on their concentration, as well as other circulating hormones concentrations such as leptin, glucocorticoids or catecholamines. These basic differences in HPT axis activity could explain the sex dimorphism in the response to stress and metabolic challenges.

An important problem is that not only women are more susceptible to subclinical and overt hypothyroidism than men, but they are also more dissatisfied with their medical treatment, expressing problems of fatigue, mood, memory and body weight gain (77, 160). A possible contributor is the stress history of the patient. Women are more susceptible than men to depression caused by chronic stress which inhibits the HPT axis. The joint treatment of stress and thyroid disorder could be more effective than the isolated medication for thyroid disfunction, particularly in modern societies that have not achieved the conditions of equality and have lost the social support of a more interactive society as probably still exists in smaller communities. Increased opportunities for exercise and cultural recreation, especially in low-income districts, could aid to reduce stress, activate metabolism and increase the feeling of wellbeing.

Finally, it should be noted that there are caveats in comparisons between sexes, between rodents and humans. Housing conditions, lack of stimuli, food *ad libitum*, and constant temperatures induce obesity and symptoms of metabolic syndrome in rodents at a young age (161). Although strain variations in the metabolic response are observed (162), most become obese at young age; Wistar male rats are obese at 2 months of age whereas females, at 6 (163). These problems complicate the interpretation of metabolic studies and make the comparison with humans very difficult. Another problem encountered within the literature is lack of information about the age of the animal and at most, their weight which depending on strain may be an adolescent or a very young animal; some animals are bought from distant places, and many are adolescents which is also a critical period when stress impacts strongly (164). Most studies about sex differences in HPT axis activity have been performed in animal models; human studies are still needed to find out how HPT axis activity is affected by stress and metabolic challenges. More holistic research is needed combining both sexes in the experimental subjects and report some parameters that may evaluate their stress and metabolic status.

## Data Availability Statement

The original contributions presented in the study are included in the article/**Supplementary Material**. Further inquiries can be directed to the corresponding author.

## Author Contributions

MP-M, IS-R, and AG-M reviewed the literature, wrote an initial draft of the manuscript, prepared tables, boxes and figures, and reviewed the final version. J-LC reviewed an initial draft and the final version. PJ-B reviewed the literature and edited the manuscript. All authors contributed to the article and approved the submitted version.

## Funding

Our work was supported by grants from DGAPA-UNAM IN213419 (PJ-B) and IN206712 (J-LC); CONACYT 0284883 (PJ-B) and PN562, CB254960 (J-LC). MP-M and AG-M were or are recipient of a CONACYT postgraduate scholarship (MP-M, 273496, AG-M, 790605); both were also recipient of a DGAPA-UNAM scholarship (IN213419).

## Conflict of Interest

The authors declare that the research was conducted in the absence of any commercial or financial relationships that could be construed as a potential conflict of interest.

## Publisher’s Note

All claims expressed in this article are solely those of the authors and do not necessarily represent those of their affiliated organizations, or those of the publisher, the editors and the reviewers. Any product that may be evaluated in this article, or claim that may be made by its manufacturer, is not guaranteed or endorsed by the publisher.

## Abbreviations

Note: italics are used for genes or mRNA names (i.e., *Trh*), and capital letters for proteins (i.e., TRH). α-MSH, α-melanocyte stimulating hormone; AgRP, Agouti-related protein; Arc, arcuate nucleus; BAT, brown adipose tissue; BMI, body mass index; CRE, cAMP-response element; CREB, cAMP-response element binding protein; CRH, corticotropin releasing hormone; Dio1, deiodinase type 1; Dio2, deiodinase type 2; Dio3, deiodinase type 3; FR, food restriction; GC, glucocorticoids; GD, Grave’s disease; GR, glucocorticoid receptor; HFCD, high unsaturated fat and high carbohydrate diet; HFD, high fat diet; HPA, hypothalamus-pituitary-adrenal; HPT, hypothalamus-pituitary-thyroid; HT, Hashimoto’s thyroiditis; MBH, mediobasal hypothalamus; MC4R, melanocortin 4 receptor; MCT, monocarboxylate transporter; ME, median eminence; MS, maternal separation; NH, non-handled; NPY, neuropeptide Y; OATP, organic anionic transport protein; OVX, ovariectomy; POMC, proopiomelanocortin; PVN, hypothalamic paraventricular nucleus; RXR, retinoid X receptor; SULT, sulfotransferase; T3, 3,5,3’-triiodothyronine; FT3, free T3; rT3, reverse T3, or 3,3’,5’-triiodothyronine; TT3, total T3; T4, thyroxine; FT4, free T4; TT4, total T4; TBG, thyroxine-binding globulin; TGB, thyroglobulin; TH, thyroid hormones; THR, thyroid hormone receptor; TPO, thyroid peroxidase; TRH, thyrotropin releasing hormone; TRH-DE, TRH-degrading ectoenzyme; TRH-R1, TRH receptor type 1; TSH, thyrotropin; TSH-R, TSH receptor; UGT, UDP-glucuronosyltransferase; WAT, white adipose tissue; XCI, X chromosome inactivation.
