# Supplementary material for: Sex Dimorphic Responses of the Hypothalamus-Pituitary-Thyroid Axis to Energy Demands and Stress
Source: Front Endocrinol (Lausanne). 2021 Oct 20;12:746924. doi: 10.3389/fendo.2021.746924 (PMC8565401; doi:10.3389/fendo.2021.746924)
Supplement: Supplementary file 1 [file DataSheet_1.docx]

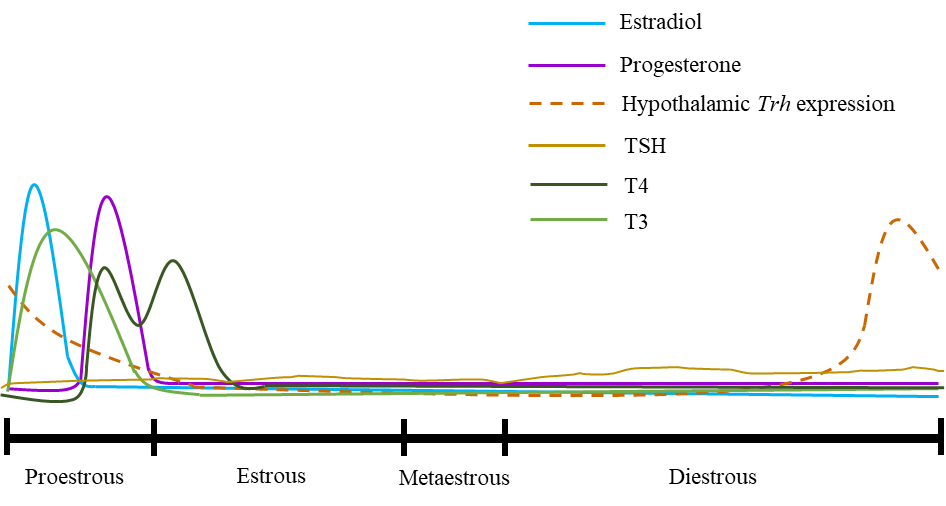


**Supplementary Figure 1.** **Profiles of estradiol, progesterone, TSH, T4 and T3 serum concentration, and hypothalamic *Trh* expression during the estrous cycle of rats.** The estrous cycle of rats lasts four days approximately and is divided in four phases: proestrus (12-14 h), estrous (25-27 h), metaestrus (6-8 h) and diestrus (55-57 h), being the first one when serum estradiol and progesterone concentrations reach their maximum value, favoring ovulation (1). These changes in the concentration of hormones may produce different physiological responses depending of cycle phase, which make it difficult to study these responses in females. HPT axis activity varies depending of phase of estrous cycle. In later diestrus phase, *Trh* expression in hypothalamus is high, and tends to decrease during proestrus and early estrous (2). Serum TSH concentration does not change during the estrous cycle (3,4,5). Serum T4 concentration has two peaks between proestrus and estrus phases, while serum T3 concentration reaches its maximum at proestrus (3).

1. Paccola CC, Resende, CG, Stumpp T, Miraglia SM, Cipriano I. The Rat Estrous Cycle Revisited: A Quantitative and Qualitative Analysis. *Anim Reprod* (2013) 10, 677-683. https://www.animal-reproduction.org/article/5b5a6046f7783717068b467f

2. Uribe RM, Joseph-Bravo P, Pasten J, Ponce G, Méndez M, Covarrubias L, Charli JL. Some Events of Thyrotropin-Releasing Hormone Metabolism are Regulated in Lactating and Cycling Rats. *Neuroendocrinology* (1991) 54:493-498. doi: 10.1159/000125943

3. Tohei A, Imai A, Watanabe G, Taya K. Influence of Thiouracil-induced Hypothyroidism on Adrenal and Gonadal Functions in Adult Female Rats. *J Vet Med Sci* (1998) 60:439-46. doi: 10.1292/jvms.60.439

4. Ayala C, Pennacchio GE, Soaje M, Carreño NB, Bittencourt JC, Jahn GA, et al. Effects of Thyroid Status on NEI Concentration in Specific Brain Areas Related to Reproduction during the Estrous Cycle. *Peptides* (2013) 49:74-80. doi: 10.1016/j.peptides.2013.08.016

5. Kieffer JD, Mover H, Maloof F. Plasma TSH levels, by Radioimmunoassay, during the Estrous Cycle of the Rat. *Endocrinology* (1975) 96:535-7. doi: 10.1210/endo-96-2-535
