# Supplementary material for: Sex Dimorphic Responses of the Hypothalamus-Pituitary-Thyroid Axis to Energy Demands and Stress
Source: Front Endocrinol (Lausanne). 2021 Oct 20;12:746924. doi: 10.3389/fendo.2021.746924 (PMC8565401; doi:10.3389/fendo.2021.746924)
Supplement: Supplementary file 2 [file DataSheet_2.docx]

**
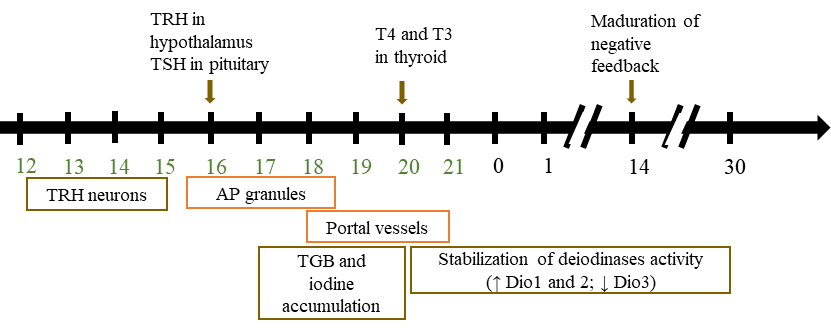
**

**Supplementary figure 2. Development of HPT axis in rats is independent of sex**. Hypophysiotropic neurons of PVN are born just before and within the last week of gestation; TRH is detected in the hypothalamus between embryonic day (E) 16 and E17, whereas TSH is detectable in pituitary at E16, but the secretion of these hormones is poor during the first postnatal days because portal vessels are not functional before postnatal day (P) 5 (1). Thyroid gland is mature at E17, and between E17 and E20 accumulates thyroglobulin and iodine (2). The activity of Dio3 is high during fetus development, limiting T3 action; after birth, Dio3 activity decreases and those of Dio1 and Dio2 increase, starting at E20, and stabilizing until P30 (3,4). The negative feedback that controls HPT axis activity matures around P14 (5).

1. Glydon RS. The Development of the Blood Supply of the Pituitary in the Albino Rat, with Special Reference to the Portal Vessels. *J Anat* (1957) 91:237-244. PMID: 13416129

2. Rémy L, Michel-Bechet M, Athouel-Haon AM, Magre S, Cataldo C, Jost A. Development of the Thyroid Gland in the Rat Fetus in vivo. An Ultrastructural and Radioautographic Study. *Arch Anat Microsc Morphol Exp* (1980) 69:91-108. PMID: 7447451

3. Bates JM, St. Germain DL, Galton VA. Expression Profiles of the Three Iodothyronine Deiodinases, D1, D2, and D3, in the Developing Rat. *Endocrinology* (1999) 140:884-851. doi: 10.1210/endo.140.2.6537

4. Hernandez A, Martinez ME, Ng L, Forrest D. Thyroid Hormone Deiodinases: Dynamic Switches in Developmental Transitions. *Endocrinology* (2021) 162:1-15. doi: 10.1210/endocr/bqab091

5. Walker P, Dubois JD, Dussault JH. Free thyroid hormone concentrations during postnatal development in the rat. *Pediatr Res* (1980) 14:247-9. doi: 10.1203/00006450-198003000-00014
