# Supplementary material for: Sex Dimorphic Responses of the Hypothalamus-Pituitary-Thyroid Axis to Energy Demands and Stress
Source: Front Endocrinol (Lausanne). 2021 Oct 20;12:746924. doi: 10.3389/fendo.2021.746924 (PMC8565401; doi:10.3389/fendo.2021.746924)
Supplement: Supplementary file 3 [file Table_1.docx]

| Region/Tissue | Model | Sex steroid treatment | Sex | Effect | References |
| --- | --- | --- | --- | --- | --- |
| Hypothalamic PVN | Rat (GDX) | E | ♀ | ↓ TRH release | (1) |
|  | Rat (GDX) | E | ♀ | ↓ *Trh* expression | (2) |
|  | Rat (GDX) | T | ♂ | ↑ TRH processing from TRH-Gly and TRH release | (3) |
| Hypophyseal portal plasma | Rat (GDX) | E/P | ♀ | ↑ TRH concentration | (4) |
| Anterior pituitary | Sheep (primary cell culture) | E | ♀ | ↑ TSH intracellular concentration and release | (5) |
|  | Rat | E | Both | ↑ TRH binding | (6) |
|  | GH_3_ and GC cell lines | E | - | ↑ TRH binding  ↑ TRH receptor density | (7) |
|  | Rat (GDX) | E | ♀ | ↑ Dio1 activity | (8,9) |
|  | Rat (GDX) | E | ♂ | ↑ Dio2 activity  ↑ Dio1 activity  ↑ T3 receptor density  ↑ TRH receptor density  ↓ TRH-DE activity | (10,11) |
|  | Rat (GDX) | P | ♀ | ↓ Dio1 activity | (9) |
|  | Rat (GDX) | T | Both | ♂: ↑ TRH sensitivity  ↑ TSHβ mRNA level  ↑ TSH secretion  ↑ Dio1 activity  ♀: No effect | (12,14,9) |
|  | Rat (GDX, old) | T | ♂ | ↑ TSHβ peptide | (15) |
| Thyroid gland | Rat (HPX or GDX) | E | ♀ | ↑ Iodine catching (HPX and GDX)  ↓ TGB hydrolysis (only GDX)  ↑ TPO activity (only GDX)  ↑ Dio1 activity (only GDX) | (16,17,8,18,19) |
|  | Rat (GDX, old) | E | ♂ | ↓ Tg-T4 | (15) |
|  | Rat (GDX; prepuber and adult) | E | Both | ♂: - TSH-R (both ages)  ♀: ↑ TSH-R (both ages) | (20,21) |
|  | Rat (GDX) | P | ♀ | ↓ *Dio2* mRNA | (22) |
|  | Human (Thyroid follicular cells) | P | - | ↑ TPO, TGB and NIS mRNA | (23) |
|  | Rat (GDX; prepuber and adult) | T | Both | ♂: ↑ TSH-R (both ages)  ♀: ↑ TSH-R (only prepuberty) | (20,21) |
|  | Rat (GDX, 16-month-old) | T | ♂ | ↑ TGB-T4 | (15) |
| Liver | Rat (GDX) | E | ♀ | - *Dio1* mRNA  ↑ Dio1 activity | (24,9,19) |
|  | Rat (GDX) | P | ♀ | ↓ Dio1 activity  ↓ *Dio2* mRNA | (9,22) |
|  | Rat (GDX) | T | ♂ | ↑ *Dio1* mRNA  ↑ Dio1 activity | (24,9,19, 15) |
| Blood | Rat (young and old) | E | ♀ | Young: ↑ serum TSH and T3 concentrations  Old: ↑ TSH, ↓ T4 and T3 serum concentrations | (25, 20,15,19) |
|  | Rat (GDX) | E/P | ♀ | ↑ TSH concentration (both hormones)  ↑ T3 concentration (only E)  ↓ T4 concentration (only E) | (3,20,2,26) |
|  | Rat (GDX) | P | ♀ | ↑ FT4 serum concentration  ↓ FT3 serum concentration  ↑ T4/T3 ratio | (22) |
|  | Rat (GDX) | P | Both | ♂: - TSH, T4 or T3 serum concentrations  ♀: ↑ serum T4 and T3 concentrations | (27) |
|  | Rat (GDX) | T | ♂ | ↑ serum TSH concentration | (25,28,20) |

**Supplementary Table 1.** **Effect of sex steroids at different levels of the hypothalamus-pituitary-thyroid axis according to model and sex.** GDX: gonadectomized; HPX: hypophysectomized; E: treatment with estradiol; T: treatment with testosterone; P: treatment with progesterone; ↑: stimulatory effect; ↓: inhibitory effect; -: without observable effects.

References:

1. Wang PS, Huang SW, Tung YF, Pu HF, Tsai SC, Lau CP et al. Interrelationship Between Thyroxine and Estradiol on the Secretion of Thyrotropin-Releasing Hormone and Dopamine into Hypophysial Portal Blood in Ovariectomized-Thyroidectomized Rats. *Neuroendocrinology* (1994) 59:202-207. doi: 10.1159/000126660
2. Uribe RM, Zacarias M, Corkidi G, Cisneros M, Charli JL, Joseph-Bravo P. 17β-Oestradiol Indirectly Inhibits Thyrotrophin-Releasing Hormone Expression in the Hypothalamic Paraventricular Nucleus of Female Rats and Blunts Thyroid Axis Response to Cold Exposure. *J Neuroendocrinol* (2009) 21:439-48. doi: 10.1111/j.1365-2826.2009.01861.x
3. Ross DS. Testosterone Increases TSH-beta mRNA and Modulates alpha-Subunit mRNA Differentially in Mouse Thyrotropic Tumor and Castrate Rat Pituitary. *Horm Metab Res* (1990) 22(3):163-9. doi:10.1055/s-2007-1004875
4. Huang SW, Tsai SC, Tung YF, Wang PS. Role of Progesterone in Regulating the Effect of Estradiol on the Secretion of Thyrotropin-Releasing Hormone and Dopamine into Hypophysial Portal Blood in Ovariectomized Rats. *Neuroendocrinology* (1995) 61:536-541. doi: 10.1159/000126877
5. Miller WL, Knight MM, Gorski J. Estrogen Action *in vitro*: Regulation of Thyroid Stimulating and Other Pituitary Hormones in Cell Cultures. *Endocrinology* (1977) 101:1455-1460. doi: 10.1210/endo-101-5-1455
6. De Leán A, Ferland I, Drouin J, Kelly PA, Labrie F. Modulation of Pituitary Thyrotropin Releasing Hormone Receptor Levels by Estrogens and Thyroid hormones. *Endocrinology* (1977) 100:1496-504. doi: 10.1210/endo-100-6-1496
7. Gershengorn MC, Marcus-Samuels BE, Geras E. Estrogens Increase the Number of Thyrotropin-Releasing Hormone Receptors on Mammotropic Cells in Culture. *Endocrinology* (1979) 105:171-176. doi: 10.1210/endo-105-1-171
8. Lisbôa PC, Curty FH, Moreira RM, Pazos-Moura CC. Effects of Estradiol Benzoate on 5’-Iodothyronine Deiodinase Activities in Female Rat Anterior Pituitary Gland, Liver and Thyroid Gland. *Braz J Med Biol Res* (1997) 30:1479-1484. doi: 10.1590/S0100-879X1997001200016
9. Lisbôa PC, Curty FH, Moreira RM, Oliveira KJ, Pazos-Moura CC. Sex Steroids Modulate Rat Anterior Pituitary and Liver Iodothyronine Deiodinase Activities. *Horm Metab Res* (2001) 33:532-535. doi: 10.1055/s-2001-17211
10. Donda DS. Testosterone Increases TSH-β mRNA and Modulates α-subunit mRNA Differentially in Mouse Thyrotropic Tumor and Castrate Rat Pituitary. *Horm Metab Res* (1990) 22:163-169. doi: 10.1055/s-2007-1004875
11. Schomburg L, Bauer K. Regulation of the Adenohypophyseal Thyrotropin-Releasing Hormone-Degrading Ectoenzyme by Estradiol. *Endocrinology* (1997) 138:3587-3593. doi: 10.1210/endo.138.9.5372
12. Christianson D, Roti E, Vagenakis AG, Braverman LE. The Sex-Related Difference in Serum Thyrotropin Concentration is Androgen Mediated. *Endocrinology* (1981) 108:529-535. doi: 10.1210/endo-108-2-529
13. Pekary AE,Knoble M, García NH, Bhasin S, Hershman JM. Testosterone Regulates the Secretion of Thyrotrophin-Releasing Hormone (TRH) and TRH Precursor in the Rat Hypothalamic-Pituitary Axis. *J Endocrinol* (1990) 125:263-270. doi: 10.1677/joe.0.1250263
14. Borges PP, Curty FH, Pazos-Moura CC, Moura EG. Effect of Testosterone Propionate Treatment on Thyrotropin Secretion of Young and Old Rats *in vitro*. *Life Sci* (1998) 62:2035-2043. doi: 10.1016/S0024-3205(98)00175-1
15. Šošić-Jurjević B, Filipović B, Milošević V, Nestorović N, Manojlović-Stojanoski M, Brkić B et al. Chronic Estradiol Exposure Modulates Thyroid Structure and Decreases T4 and T3 Serum Levels in Middle-Aged Female Rats. *Horm Res* (2005) 63:48-54. doi: 10.1159/000083139
16. Boccabella AV, Alger EA. Influence of Estradiol on Thyroid: Serum Radioiodine Concentration Ratios of Gonadectomized and Hypophysectomized Rats. *Endocrinology* (1964) 74:680–688. doi: 10.1210/endo-74-5-680
17. Bagchi N, Shivers B, Brown TR. Effects of Castration and Sex Steroids on the Thyroid Response to Thyrotropin. *Endocrinology* (1984) 114:1652-1656. doi: 10.1210/endo-114-5-1652
18. Lima LP, Barros IA, Lisbôa PC, Araújo RL, Silva AC, Rosenthal D et al. Estrogen Effects on Thyroid Iodide Uptake and Thyroperoxidase Activity in Normal and Ovariectomized Rats. *Steroids* (2006) 71:653-659. doi: 10.1016/j.steroids.2006.03.007
19. Marassi MP, Fortunato RS, da Silva AC, Pereira VS, Carvalho DP, Rosenthal D et al. Sexual Dimorphism in Thyroid Function and Type 1 Iodothyronine Deiodinase Activity in Pre-Pubertal and Adult Rats. *J Endocrinol* (2007) 192:121-130. doi: 10.1677/joe.1.06901
20. Banu SK, Arosh JA, Govindarajulu P, Aruldhas MM. Testosterone and Estradiol Differentially Regulate Thyroid Growth in Wistar Rats from Immature to Adult Atage. *Endocr Res* (2001) 27:447-463. doi: 10.1081/ERC-100107868
21. Banu SK, Govindarajulu P, Aruldhas MM. Testosterone and Estradiol Modulate TSH-Binding in the Thyrocytes of Wistar Rats: Influence of Age and Sex. *J Steroid Biochem Mol Biol* (2001) 78:329–342. doi: 10.1016/S0960-0760(01)00107-8
22. Awad HA, Alrefaie ZA. An Evidence for the Transcriptional Regulation of Iodothyronine Deiodinase 2 by Progesterone in Ovariectomized Rats. *J Physiol Biochem* (2014) 70:331-339. doi: 10.1007/s13105-013-0307-y
23. Bertoni APS, Brum IS, Hillebrand AC, Furlanetto TW. Progesterone Upregulates Gene Expression in Normal Human Thyroid Follicular Cells. *Int J Endocrinol* (2015) 2015:1-6. doi: 10.1155/2015/864852
24. Miyashita K, Murakami M, Iriuchijima T, Takeuchi T, Mori M. Regulation of Rat Liver Type 1 Iodothyronine Deiodinase mRNA Levels by Testosterone. *Mol Cell Endocrinol* (1995) 115:161-167. doi: 10.1016/0303-7207(95)03689-X
25. Chen HJ. Age and Sex Difference in Serum and Pituitary Thyrotropin Concentrations in the Rat: Influence by Pituitary Adenoma. *Exp Gerontol* (1984) 19:1-6. doi: 10.1016/0531-5565(84)90025-1
26. Pantaleão TU, Mousovich F, Rosenthal D, Padrón AS, Carvalho DP, Correa da Costa VM. Effect of Serum Estradiol and Leptin Levels on Thyroid Function Food Intake and Body Weight Gain in Female Wistar Rats. *Steroids* (2010) 75:638-642. doi: 10.1016/j.steroids.2010.03.009
27. Jeyaraj DA, Maran RRM, Aruldhas MM, Govindarajulu P. Progesterone Induced Modulations of Serum Hormonal Profiles in Adult Male and Female Rats. *Endocr Res* (2001) 27:223-232. doi: 10.1081/ERC-100107183
28. Borges PP, Curty FH, Pazos-Moura CC, Moura EG. Effect of Testosterone Propionate Treatment on Thyrotropin Secretion of Young and Old Rats *in vitro*. *Life Sci* (1998) 62:2035-2043. doi: 10.1016/S0024-3205(98)00175-1
